# Benefits of Advanced Practice Nursing for Its Expansion in the Spanish Context

**DOI:** 10.3390/ijerph16050680

**Published:** 2019-02-26

**Authors:** María Begoña Sánchez-Gómez, Sara Ramos-Santana, Juan Gómez-Salgado, Francisca Sánchez-Nicolás, Carlos Moreno-Garriga, Gonzalo Duarte-Clíments

**Affiliations:** 1 Nursing School Nuestra Señora de Candelaria, University of La Laguna, 38010 Santa Cruz de Tenerife, Spain; begonasanchez@gmail.com (M.B.S.-G.); gonzaloduartecliments@gmail.com (G.D.-C.); 2 Primary Care Management of Tenerife, Canary Islands Health Service, 38200 San Cristóbal de La Laguna, Spain; sari-rs@hotmail.com (S.R.-S.); mediterraneapsn@gmail.com (F.S.-N.); cmorgar@gobiernodecanarias.org (C.M.-G.); 3 Nursing Department, University of Huelva, 21007 Huelva, Spain; 4 Safety and Health Posgrade Program, Universidad Espíritu Santo, Samborondón, 091650 Guayaquil, Ecuador

**Keywords:** advanced practice nursing, evidence-based practice, managed care, nursing practice, quality of care, research utilisation

## Abstract

The objective of this study is to describe the impact of the Advanced Practice Nurse role on the clinical practice and patient benefit, as well as to provide reasons for its implementation and expansion in Spain. Through the scoping review method, this study has been carried out according to five thematic blocks: life quality, cost-effectiveness, health results, satisfaction, and accessibility. The critical appraisal was performed with the Critical Appraisal Skills Programme (CASP) tool and the level of evidence and strength of recommendation have been analysed following the Oxford Centre for Evidence-Based Medicine (OCEBM) system. The results show that it is possible to formally implement advanced practice nursing in the Spanish context. The analysis of the Spanish regulatory framework reveals that the generalisation of the Case Manager Nurse is the starting point for the development of advanced practice nursing in Spain. This implementation would have a positive impact on patients in terms of health results, satisfaction, and life quality, given that the advanced practice nurse performs a more effective follow-up of chronic patients with a better control of risk factors, symptoms and health outcomes, and an earlier detection of complications. Considering these results, regional governments should promote the role of the Advanced Practice Nurse to contribute to its expansion.

## 1. Introduction

The International Council of Nurses defines the Advanced Practice Nurse (APN) as “a registered nurse who has acquired the expert knowledge base, complex decision-making skills and clinical competencies for expanded practice, the characteristics of which are shaped by the context and/or country in which s/he is credentialed to practice” [1].

A master’s degree is recommended for entry level, so the APN has educational preparation at an advanced level, formal recognition of educational programmes training nurse practitioners/advanced practice nurses that are accredited or approved, and a formal system of regulation, accreditation, registration, certification, and credentialing. The APN integrates research, education, practice, and management, has a high level of professional autonomy, advanced health assessment skills, decision-making skills, and diagnostic reasoning skills. In addition, the APN plans, implements, and evaluates health programmes. The APN is also known for having recognised advanced clinical competencies and providing consultant services to other health providers [1,2,3]. The professional practice of the APN comes from a reflective practice based on a wide range of knowledge skills obtained from nursing and other disciplines, which can be an entry point to the health system. Some expressions of this practice model imply the capacity to prescribe, to refer patients, or to admit patients in institutions [4]. 

As society evolves, population’s health needs are more complex. Population ageing, the increase of chronic and disabling diseases, and patients’ greater expectations make it necessary to find new ways of globally addressing this phenomenon. Assisting chronic patients constitutes one of the main challenges for all health systems. In Spain, designing and applying strategies to address this situation is a matter of concern derived from the progressive ageing of the population, as it implies high economic costs for health services and Regional Governments [5].

In order to deal with this new and complex health demand of the population, reforms in health systems strategies include increasing the number of nurses working in clinical settings or advanced practice and expanding their roles. The analysis of the experiences of other countries where the APN programmes have been developing for years can serve as a reference in the attempt to integrate advanced practice into the Spanish context.

### 1.1. Background

The role of the APN was described for first time in the United States in the 1970s as the Advanced Practice Registered Nurse (APRN). This term replaced the previous “specialisation” and was defined for four different nursing fields of action: midwife, nurse anaesthetist, clinical nurse specialist, and nurse practitioner [6]. The two first professional roles related to the APN in the United States emerged in response to different needs. On the one hand, the Nurse Practitioner appeared to cover a lack of medical assistance in rural areas due to shortage of physicians. The advanced practice of these nurses was based on assessment, diagnosis and treatment skills, which gave them autonomy in the management of certain patients. On the other hand, the Clinical Nurse Specialist met the increasing population’s demands and the need for nurse managers to have someone to help them train their staff in quality of care. Therefore, the advanced practice of these nurses has been based on their own nursing care. Recently, it stands out as a formula for more cost-effective organisational systems that guarantee the access to quick, coordinated, and qualified services for all users [7].

Later, in the 1990s, in Canada, the APN was included in the post-graduate and doctorate nursing studies, represented by Nurse Practitioners [8]. Gradually, the APN role has been developed in other countries such as the United Kingdom, Australia, New Zealand, Holland, Sweden, and Ireland [9]. The development of APN was adapted to the particularities of each context, leading to the heterogeneity of conceptions. In an effort to establish a definition, the International Council of Nurses defined the APN as a nurse with an expert knowledge with the ability to make complex decisions necessary to carry out an extended practice [7].

### 1.2. International Context

In the 2010 working paper of the Organisation for Economic Cooperation and Development (OECD), the development of APN in 12 countries was monitored and analysed from its origin to date: Australia, Belgium, Canada, Cyprus, Czech Republic, Finland, France, Ireland, Japan, the United Kingdom, and the Unites States [10]. The common factors presented by most of these countries for the development of the APN were motivated by the following issues:

- Shortage of primary care physicians, mainly in remote and rural areas.
- Constant changes in the population’s health needs related to the increase of chronic diseases and population ageing.
- The need for improving the quality and continuity of assistance.
- To improve career prospects for nurses.

For these reasons, the reforms made by these countries in their health systems included scaling-up strategies for the nursing role and an increase in the number of workers in advanced levels. The factors that contributed to the development of APN in most countries were the following:

- Demand from the nursing associations.
- Patient support.
- Governments’ support by means of legislative changes and financing.
- Education systems’ capacity to train nurses for more advanced roles.
- Positive attitude of health managers.

In contrast, the barriers identified by the OECD were the opposition of medical associations as well as legislation, as it limits the implementation of APN, for instance, regarding drugs prescription in some countries.

This analysis of facilitators and barriers is consistent with what was described by Goodman et al. [11]. They described facilitators such as the development of nursing specialties, restructuring of the National Health System, effectiveness assessment of nursing interventions, and a framework for professional competence training. As barriers, the authors identified difficulties in the conceptualisation and regulation of the APN, in professional interests, in characteristics of the organisation of the services offered, and in legislation. A summary of the characteristics found in the OECD report of countries is shown in Table 1.

APN functions are similar among countries: evaluation and diagnosis (both physical and psychological), assistance of acute health problems and management of chronic diseases, emergency triage, health education, counselling, research, and leadership management. The meta-analysis conducted by Hutchinson et al. revealed seven domains of advanced nursing practice: autonomous or nurse-led extended clinical practice; improving systems of care; developing the practice of others; developing/delivering educational programmes/activities; nursing research/scholarship; leadership external to the organisation; and administering programmes, budgets, and personnel [12]. Hamric et al. identified the following APN skills: expert clinical practice, guidance and coaching, consultation, evidence-based practice, clinical and professional leadership, collaboration, and ethical decision-making [13].

### 1.3. Spanish Context

In Spain, we find a lack of definition of the APN field of action. Many Regional Health Governments have launched new nursing roles with differentiated profiles and extended competences. These new nursing profiles can be considered as advanced practices, and they meet patients’ demands in complex situations derived from chronicity, comorbidity, weakness, and ageing. In the Canary Islands, the role of the community liaison nurse has been developed since 2000, which falls within the so-called continuity of care at home service. The Basque Country has the role of the Hospital Liaison Nurse Manager, the Nurse Continuity Manager, and the Nurse Manager of Advanced Competencies. They were developed as a result of the implementation of a strategy for addressing chronicity. In Andalusia, the Case Manager Nurse (CMN) was stablished to help families in coping with complex health situations, including two specific roles: a community CMN and a hospital CMN. This role was also implemented in Catalonia, Valencia, Navarra, Madrid, Murcia and Aragon. According to the APN competencies and functions mentioned before, the CNM could be considered as an APN [5].

The CMN is the role most often used by Regional Governments in addressing the complex health situations of chronic patients and their care needs. Although it is officially recognised in nine out of the 17 Regional Health Services, it follows a disparate implementation as guideline [5,6,11,14].

For society to be able to address the provision of care, in the light of the increasing current complexity, it is necessary to invest in postgraduate nurses with a high level of clinical training and leadership [11]. The university of Navarra (Spain) offers a master’s degree course in Advanced Clinical Nurse since 2004. The training programme trains nurses in APN competencies, and it is leading the implementation and development of the APN role at a national level [15,16,17]. There is an increasing number of nurses who prove to be cost-effective and as decisive as health professionals. These nurses develop case management roles or other roles that have been incorporated into the management of complex chronic patients and caregivers [8].

### 1.4. APN Competencies

The World Health Organization (WHO) and the International Council of Nurses have identified seven key areas of competence of the APN, focused on: health and patients’ diseases management, relationship with patients, coach-teacher role, professional role, use and management of the system of provision of care services, monitoring and quality guaranteeing of the clinical practice, and cultural care [2].

In 2017, these competences were reviewed and updated by the National Organization of Nurse Practitioner Faculty (NONPF), establishing nine areas of competence for the APN: scientific foundation, leadership, quality, practice inquiry, technology and information literacy, policy, health delivery system competencies, ethics, and independent practice [18].

Delamaire and Lafortune underlined the role of the APN in Primary Care (PC) for providing a range of services such as the first contact with patients with minor and acute problems, along with chronic patients’ follow-up, prescription of medicines, and request for diagnostic tests [9].

The APN responsibilities in PC can be described according to the four competences inherent to the definition of the role [2]:

- Advanced Clinic: patients’ first contact with the system, either by telephone assistance, emergencies, or planned appointments for patients with minor health problems. Some of these demands can be answered with health advice or specific indications, or with the prescription of drugs. In other more acute cases, the need for other diagnostic tests, further monitoring of the process, or referral to other professionals may be needed. The APN can also interpret the results and make clinical judgments both autonomously and jointly with other team members. This offers high autonomy in the chronic patient management and avoids unnecessary referrals and potential complications. The prescribing authority allows the APN to start a specific treatment or renew a previous one within their specific group of patients.
- Management: a management procedure that allows the APN to refer to socio-health professionals, and vice versa.
- Teaching: active participation in training programmes for nursing students and registered nurses.
- Research: evidence-base facilitator, elaborating evidence-based recommendations, protocol development and clinical practice guidelines, and taking part in the design of quality standards and indicators, and in patients’ assessment and safety.

Considering all the above, the objective of this study is to describe the impact of APN in clinical practice and patient benefit, to provide evidence that serves as an argument for its implementation and expansion in Spain, particularly the role of the nurse case manager in Primary Care.

## 2. Materials and Methods 

The authors followed the scoping review methodology described by Arksey and O’Maley. This method has a narrative and descriptive approach in order to obtain an overview and a synthesis of the available studies [19].

The databases consulted were Virtual Health Library (VHL), Medline, and Cochrane. Combinations of the following key words were used: “Advanced Practice Nurse”, “cost-benefit analysis”, “quality of life”, “cost-effectiveness”, “competence” and “implementation”. The descriptors were combined using the Boolean operator “AND”. The PICO (Population; Intervention; Comparison; Outcome) framework, considering nurse care receiver (patient), APN (intervention), standard nursing practice (comparison) and health benefits (outcome) was used as a tool for formulating research questions.

The search was performed both in English and in Spanish from January to May 2015. All researchers participated individually, sharing and comparing outcomes.

The inclusion criteria were research works on APN published in the last fifteen years whose results related to health outcomes, quality of life, cost-effectiveness, service satisfaction, and health service accessibility, attributable to nursing interventions. The critical appraisal was performed with the Critical Appraisal Skills Programme (CASP) tool according to the design, selecting those publications with a positive answer to five questions or more (including the two first questions), in order to guarantee a high methodological quality [20].

Recommendations obtained from the articles reviewed were classified according to their level of evidence and strength of recommendation following the Oxford Centre for Evidence-Based Medicine (OCEBM) system. This proposal is characterised by assessing the evidence according to the thematic area or clinical scenario and the type of study that involves the clinical problem in question. This classification prioritises evidence at levels ranging from 1 to 5, being level 1 the “best evidence” and level 5 the “least good”. It also identifies four grades of recommendation where A corresponds to the strongest recommendation and D to the weakest.

The analysis was performed by a peer review, and discrepancies were resolved by consensus among the research team. The excluded studies were those which did not meet the inclusion criteria or obtained a result lower than 5 in the critical appraisal.

The data extracted was organised according to the following criteria: health outcomes, life quality, cost-effectiveness, satisfaction with the provision of the service, and accessibility to the health system, and results were summarised in a narrative way. 

## 3. Results

**Figure 1:**
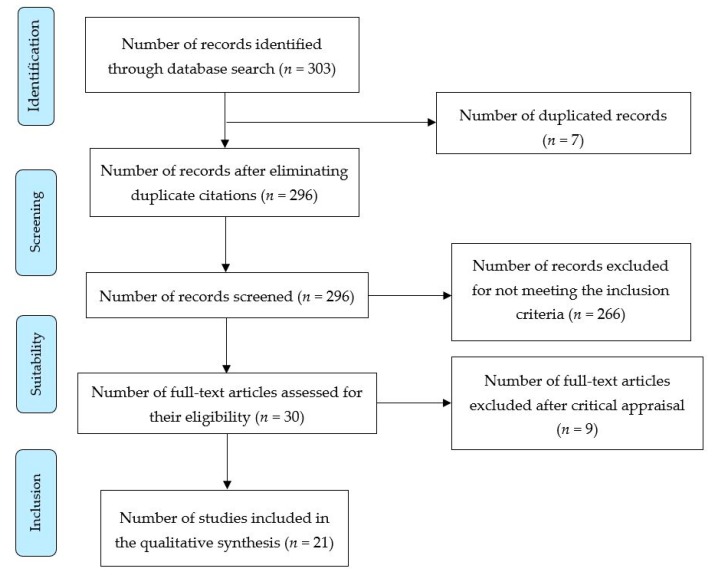
Flow chart of the search strategy.

The initial electronic search produced 303 references. After eliminating duplicates, 296 records were selected for screening. Out of these, 30 full-text articles were selected for eligibility criteria. Finally, 21 studies were selected for this review. Figure 1 shows the flowchart of the selected studies, following the PRISMA (Preferred Reporting Items for Systematic Reviews and Meta-Analyses) recommendations [21].

According to the methodology, the present review includes eight systematic reviews, seven qualitative articles with narrative review format, five clinical experiments, and one case and control article. Data on the results obtained by the authors were selected from each study. The main findings of the studies are collected in Table 2.

The recommendations for clinical practice were extracted from the results of the studies reviewed. Most levels of evidence correspond to the highest level, although they range from 1 to 5. The degree of recommendation remains between A and B, as shown in Table 3. As a narrative summary, it is observed that, with the implementation of the APN role in different contexts and countries, numerous studies have been developed on the assessment of its impact from different perspectives, which gives evidence of the APN contribution to the health system and to the assisted patients.

### 3.1. Quality of Life

The research carried out by Laurant et al. assessed the workload perceived by other health professionals, as well as other organisational aspects subsequent to the introduction of the APN in the Netherlands. The results showed a meaningful decrease of the emergency service demand after the creation of an APN clinic in PC, although no significant differences in workload were found for family physicians [22].

Another study also showed a slight increase of APN consultation demand by some patients, mostly those who suffered from asthma or chronic obstructive pulmonary disease. This seems to be derived from the APN capacity to detect health problems or potential complications not previously studied, highlighting that the APN contributes to chronic patients’ assistance as a significant qualitative element [23,24].

Also, in 2013, the systematic review by Donald et al. on the effectiveness of advanced practice nurses in long-term care included four prospective studies conducted in the United States. The findings suggested that the APN provision of care improved health and quality of life in nursing home residents. Moreover, their families were more satisfied with the attention received. Nursing homes showed lower rates of depression, urinary incontinence, pressure ulcers, restraint use, and aggressive behaviour. At the same time, residents experienced improvements that helped achieving their personal goals [25].

### 3.2. Cost-Effectiveness

In the PC context, APN can successfully meet the systems’ demand in terms of outcomes and referrals, and as compared to family physicians. Some researchers have identified qualitative and quantitative differences between the services provided by family physicians and by APN in PC. The APN spent more time in each consultation, with a higher awareness of providing information to the patient and their family about the illness and the best therapeutic management possible, through individualised attention [1,3,22,26]. This new organisational model tends to balance health care load, improve users’ access to the health services through the diverse entry points, and to potentially reduce the expenditure derived from the lack of efficiency [1,13,31]. When attended by an APN, health problems were earlier detected and, as a result, shorter hospital stays, and less expenditure were identified [27].

### 3.3. Health Outcomes

The relevance of introducing APN in managing patients with chronic conditions was identified. In this line, an improvement in diabetes and hypertension control [28], and their associated risk factors [27] was found. In terms of respiratory problems such as chronic obstructive pulmonary disease and asthma, a higher control of the symptoms and a decrease of exacerbations was pointed out, although the results were not conclusive [23,24]. Other authors underlined the usefulness and added value of the introduction of APN for fragile patients or those with a high level of dependency [29,30,31]. The APN role showed results comparable to other health professionals, emphasising the APN ability to work autonomously, perform diagnosis, and make reliable and effective decisions [9,10,26,27,30,32,33].

APN was a key factor regarding patient’s improvement, thus reducing care provision expenditure. In this sense, the interpersonal and knowledge skills the APN possesses were of vital importance [27]. The implementation of APN was recommended to obtain a greater access and an improvement of the provision of care, and to reduce health assistance expenditure. The APN provided high quality, cost-effective, and personalised care. It is expected to play an important role in elderly nursing homes and to help and reinforce physicians’ practice, which will reduce hospital admissions. As evidence showed, APN will therefore reduce hospitalisation and referrals to emergency services from elderly care homes. APN can also adequately perform evaluations and interventions in the absence of a physician, which is commonly due to the high workload they have in multiple elderly care homes. The APN role shall be introduced as a PC giver in this type of centres so as to improve patients’ results and help physicians reduce their workload [30].

### 3.4. Satisfaction

The work of the APN reflected a higher patients’ satisfaction [5,9,10,22,25,33,34]. Studies on APN found a higher level of satisfaction, less prescription and referral cases, and more dedication in consultation. In addition, patients claimed they received more information. All this suggests that nurses provide high-level care to their patients and reinforce their essential role in primary care [34]. 

### 3.5. Accessibility

This new organisational model tries to balance healthcare loads, provide users with better access to the health services through the different entry points and to, consequently and potentially, reduce the costs derived from the lack of efficiency [9,26,34]. Another long-term advantage of the implementation of APN in PC is the progressive reorientation of health care demands, which means, in some cases, filling healthcare gaps, complementing existing services with an added value, or assuming some shared assistance with other professionals or independently, thus reordering the flow of the demand [9,22,26,30,32].

## 4. Discussion

The results of this review showed that the implementation of the APN has a positive impact on patients in terms of health outcomes, quality of life, satisfaction, cost-effectiveness, and accessibility to the health service. 

In general, the available evaluations of the APN role reveal that it can improve the access to services and reduce waiting times. It is also pointed out that the APN can provide the same quality care as physicians for a variety of services, for instance, routine monitoring of chronic patients or first contact with patients with minor health problems, for which they have received appropriate training. Most of the evaluations find a high rate of satisfaction in patients as for the services offered by the APN and, in some cases, even better than in those provided by physicians. This seems to be derived, mainly, from the fact that the APN tends to spend more time with each patient, giving them more information and advice.

Chronic illnesses are the most challenging issues for health services, and the conventional assistance models have clearly failed in addressing them. Many of the proposed strategies developed to face this challenge consider nurses as the main care providers. Many countries are trying to improve health assistance through a review of the health professional roles, including more APN in the majority of cases [10].

In this context and, as derived from international tendencies, in order to obtain a high quality, quick, and efficient health assistance it is necessary to redirect the existing strict organisation and to establish the adequate mechanisms to satisfy population needs. This may be done by an unquestionable redefinition of professional competence in terms of training and responsibilities. By means of using the great potential that nursing professionals have, we must insist on the imperative to maintain continuous and integrated care, especially in chronic processes follow-up.

The implementation of the APN implies assuming new roles and responsibility areas, specifically defined and institutionally recognised within the system. These will be accompanied by an economic retribution according to the competence level and a wide services portfolio to meet the population’s demands [2].

As limitations of this study, the systematic review search strategy can have a potential selection bias due to the established criteria: the selection of language and date, and the critical appraisal of articles. Also, some of APN implementation experiences in PC in Spain may not be reported in publications included in the consulted databases. Despite these limitations, evidence-based arguments have been gathered for the APN expansion in the Spanish context.

## 5. Conclusions

The recommendations that arise from this review show that APN are able to perform a more effective follow-up of chronic patients, with a better control of risk factors, symptoms and health outcomes and an earlier detection of complications. From the patients’ point of view, APN could increase quality of life, empowerment, and also patients’ and relatives’ satisfaction through longer and more detailed consultations. From the health service point of view, APN may decrease hospital admissions, reduce costs and waiting lists, support sustainability of the health services and a better health accessibility and team coordination. The evidence found provides arguments and support for the expansion of the APN in Spain. In light of these results, regional governments should promote the incipient role of the CMN. The Spanish regulatory framework considers the CMN role, so its expansion could be the starting point for the development of the APN in Spain. Therefore, the implementation of CMN should be prioritised in chronic and highly dependent patients.

## Figures and Tables

**Table 1:** Summary of APN (Advanced Practice Nurse) characteristics according to the OECD (Organisation for Economic Cooperation and Development) report [9].

Countries	Date of Implementation	Institutional Support	APN Categories	Educational Level	Diagnostic Tests Prescription Competence	Medical Prescription Competence	Specialist Referral Competence
Australia	1990s	stralian Nursing and Midwifery Council, (ANMC)	NP	Degree Master	yes	yes	yes
Belgium		No	APN	Degree Master			yes
Canada	1960s	Yes	NP/CNS	Degree	yes	yes	yes
Cyprus		Yes	Diabetic Nurse CMHN MHN Community Nurse	Postgraduate			
Czech Republic	Now	Yes	SN	Clinic Master Specialty	yes	no	
Finland		Yes	Public health nurse. Chronic patients nurse manager. Acute health issues nurse.	Postgraduate	yes		yes
France		HPST law	Expert nurse: AP/Home chemotherapy/Haemodialysis/Hepatitis C/Neuro-oncological link/Gastrointestinal examination/Blood donation.	Postgraduate Master		yes	
Ireland	1990s	NCNM legislation	CNS/APN	Specialty postgraduate Master	yes	yes	
Japan	now	yes	APN Specialist	5 years of specialty clinical practice and 3 specific years			
Poland	now	yes	APN		yes	no	
United Kingdom	1970s	yes	CNS/ANP/Nurse consultant/Modern Matrons/Community matrons	Graduate and experience in the specialty. Postgraduate Master	yes	yes	yes
USA	1960s	yes	APRN/CNS/CRNA/CNM/CNP/NP	Graduate Postgraduate	yes	yes	yes

Nursing and Midwifery Council, (ANMC); Nurse Practitioner, (NP); Clinical Nurse Specialists, (CNS); Community Mental Health Nurses, (CMHN); Mental Health Nurse, (MHN); Specialized Nurse, (SN); French Public Health Code, (HPST); National Council for the Professional Development of Nursing and Midwifery, (NCNM); Advanced Practice Nursing, (APN); Advanced Practice Registered Nurse, (APRN); Certified Registered Nurse Anesthetist, (CRNA); Certified Nurse-Midwife, (CNM); Certified Nurse Practitioner, (CNP).

**Table 2:** Review of selected articles.

Author, Year, Country, Study Design	Critical Appraisal (CASPe Score)	Aim	Results
Appleby & Camacho-Bejarano, 2014 [2], Spain, Narrative review	5	To analyse APN as a suitable strategy to obtain the best outcomes in terms of overall health and quality of life of patients with chronic conditions.	APN contribution to chronic patients’ management: health outcomes, coordination/team work, service quality, patient interaction-relation.
Morales Asencio, 2012 [3], Spain, Narrative review	5	Analysis of the different conceptual, regulative, legislative, and competence barriers, as well as organisational and professional ones, that Spanish nursing is facing in its new consolidation of roles.	Five big barriers were identified: difficulties in the conceptualisation and regulation n of the APN, professional interests, service organisational characteristics, training, and professional competitiveness. In the Spanish context, APN follows disparate guidelines of the Regional Governments.
De Pedro, 2006 [4], Spain, Qualitative study	5	Comprehensive analysis of the reality concerning the social demand of care, how social changes interfere with this demand, and of our ability to address it as a collective.	The first step must be a debate with the academic area, where a readjustment of the educational training and a redefinition of competencies must be done. All the more, care provision progressively requires a complexity that justifies advanced and specialised practice. We need leaders who are capable of combining the strategic vision, the precise knowledge of our abilities, and the society needs. The application and management of the Specialisation Law places us in the need for a regulation for advanced practice.
Sánchez-Martín, 2014 [5], Spain, Narrative review	5	To describe the need to reorganise and reinforce PC teams so that this area deals with chronic patients and their home comprehensive care.	APN is cost-effective and highly conclusive. It improves assistance quality and coordination with the social and health sector, decreases emergency admissions in complex patients with multiple illnesses, and improves assistance satisfaction.
Delamaire & Lafortune, 2010 [10], France, Systematic review	8	To review the evolution of the APN in 12 countries with a special emphasis on primary care functions. To review the assessment of the impact on patients’ care and on costs.	The APN can improve the access to services and reduce waiting times. They are able to offer the same quality care as physicians for specific types of patients, including those with minor ailments and those who need monitoring. A higher level of patients’ satisfaction.
Goodman et al., 2013 [11], Narrative review	5	To highlight the added benefits that the APN brings to both patients and their families in those centres with these resources, as opposed to those which provide traditional assistance.	The functions of APN are safe and as effective as those of physicians. In addition, they achieve a high level of patients’ satisfaction compared to that obtained by physicians. The APN services contribute to a neutral cost, facilitate hospitalisation costs and the use of the emergency services and, at the same time, improve the access to specific services.
Hernández Yáñez, 2010 [14], Spain, Narrative review	5	To contextualise and document the current situation of nursing in industrialised countries, with a particular focus on Spanish nursing.	The CPN is valued very positively, both for its rationalisation of qualified human resources, as well as for its contribution to financial efficiency and its impact on care quality and safety.
Laurant et al., 2004 [22], UK, Randomised clinical trial	10	To evaluate general physicians’ burden when adding a NP to their team.	The number of out-of-hours consultations in the NP intervention group was reduced. The addition of the NP to general medical teams reduced the workload, at least, in the short term. This implies that the NP is used as a complement, instead of as a replacement.
Kuethe, Vaessen-Verberne, Elbers & Van Aalderen, 2013 [23], Netherlands, Systematic review	9	To review the effectiveness of asthma care provided by nurses specialised in asthma, a NP, a medical assistant, or a professional nurse specialised in other areas working independently, as opposed to the traditional care given by a physician, both in hospitals and in PC.	There were no statistical differences in the number of asthma exacerbations and in the acuteness of the same after the treatment. Only one research had the outcome parameter of health costs, not finding statistically significant differences.
Taylor et al., 2005 [24], UK, Systematic review	9	To determinate the effectiveness of innovations in the development of chronic illness implying nurses’ actions with COPD patients.	Long-term interventions did not improve the patients’ health as for their psychological welfare, impairment, or lung function. A greater control of the symptoms and a decrease of the exacerbations is highlighted, although the results are not always conclusive.
Donald et al., 2013 [25], Canada, Systematic review	8	To deliver quantitative evidence of the effectiveness of the APN functions, specialists in clinical nursing, and nurses in meeting the health needs of older adults who live in long-term care homes.	Long-term care homes with APNs had lower rates of urine incontinence, pressure ulcers, and aggressive behaviour. Most residents experienced improvements in the achievement of personal goals and a higher level of satisfaction of the families. The APN is associated with improvements in several health status situations and in the behaviour of residents in long-term stays, as well as a higher level of satisfaction of the families.
Brooten et al., 2002 [27], USA, Systematic review	7	To describe the development of the Quality Cost Model of APN in transitional care of patients’ evolution and health assistance in the US for 22 years. To formulate what has been learnt about the evolution of nursing, its practice, and additional research.	Patients’ improvement and costs reduction in all groups that worked with APN. Time reduction in re-hospitalisations due to an early intervention and detection.
Ishani et al., 2011 [28], USA, Randomised clinical trial	11	To determine if the nurse case manager with a therapeutic algorithm can effectively improve the control rates for arterial hypertension, hyperglycaemia, and hyperlipidaemia, in contrast with the assistance received among the diabetes veterans.	A greater number of people assigned to case management achieved the results of having all outcome measures under control. In addition, they achieved the goals of individual treatment.
Chouinard et al., 2013 [29], UK, Cases and control study	8	Analysis of the efficiency and cost-effectiveness of the intervention in patients with chronic diseases who perform numerous visits to hospital services. This combines the management of cases by a nurse and the promotion of self-management.	The integration of a nurse case manager intervention and of the self-management group for primary care practices have the potential to positively impact on the patients’ improvement and their quality of life. The health care workload is expected to be reduced.
Joanna Briggs Institute, 2010 [30], Systematic review	9	To present the best evidence available of the APN role in elderly care homes.	Statistically significant decrease in emergency visits and lower rate of hospitalisation when the APN was integrated into the medical team. Implementation of APN as a supplier of primary care to reduce the use of acute assistance services to elderly people in elderly care homes.
Oeseburg, Wynia, Middel & Rejineveld, 2009 [31], Netherlands, Systematic review	9	To evaluate the effect of case management in the patients’ use of the health services and in health costs for older patients with mayor impairments or adults with chronic somatic illnesses that live in the community.	No research showed a relevant clinical increase of the use of services and of costs, whereas two researches showed that case management reduced the use of health services and was more cost-effective. The implementation of case management should be prioritised for patients with chronic illnesses and for older handicapped people.
Dierick-van Daele, Metsemaker, Derckx, Spreeuwenberg & Vrijhoef, 2009 [32], Netherlands, Randomised clinical trial	10	To evaluate the process and the results in the assistance provided to patients with common complaints by general nurses or specifically trained nurses (NP) as a first point of contact.	In both groups, the patients appreciated the quality of the assistance provided. There were no statistically significant differences in their health status, use of the medical resources, and the commitment to PC practical guidelines. Patients in the NP intervention group were more frequently asked to visit again, had more monitoring consultations, and these were significantly longer.
Kinnersley et al., 2000 [33], UK, Randomised clinical trial	10	To identify the differences between the NP and the general practitioner assistance for patients who ask for immediate consultations in primary care.	The NP consultations were longer and the patients confirmed to have been better assisted and informed. This is associated with a greater level of satisfaction.
Horrocks, 2002 [34], UK, Systematic review	9	To determine if NPs can provide first level contact assistance in a primary care centre.	Patients were more satisfied with the care provided by the NP. No differences were found in their health status. The NP had longer consultations and did more research than physicians. The NP quality of assistance was somehow better.

CASPe score: acceptable above 2; Primary Care, (PC); Certified Nurse Practitioner, (CNP); Chronic Obstructive Pulmonary Disease, (COPD); United States, (US); United Kingdom, (UK); Nurse Practitioner, (NP).

**Table 3:** Evidence level/Degree of recommendation synthesis.

Advantages of APN Interventions	Clinical Research				Systematic Review							Case/Control	Narrative Reviews				
	22	28	32	33	27	34	10	23	24	31	25	29	2	5	26	11	14
Improve the control of cardiovascular risk factors in diabetes patients for a year.		1c/A															
Obtain as good health outcomes and quality of assistance as physicians.	1c/A		1c/A	1b/A		1c/A	1c/A							1b/A	1c/A	1c/A	
Increase patients’ satisfaction with quality of service.	1c/A		1c/A	1b/A		1c/A	1c/A	1a/A	1a/A					1c/A	1c/A	1c/A	
Closer patients’ follow-up.	1c/A		1c/A				1c/A							1c/A		1c/A	
Longer time duration of consultations.	1c/A		1c/A				1c/A							1c/A		1c/A	
More detailed information received by patients.	1c/A		1c/A	1c/A			1c/A							1c/A		1c/A	
Positive impact on patients’ empowerment, health outcomes and quality of life.		5/B				3b/B	3b/B						5/B	5/B		5/B	
Greater control of symptoms and a decrease of exacerbations in respiratory processes.								1a/A	1a/A								
Decrease of hospital admissions, re-hospitalisation and hospital length of stay.	2b/A/B				1a/A			1a/A	1a/A	2b/A/B	2b/A/B	2b/A/B		1a/A		1a/A	
Better management ability and commitment in patients with COPD.								1a/A	1a/A					1a/A		1a/A	
Reduces costs in health services	1b/A		1b/A	1b/A	1a/A		1a/A		1a/A	1a/A	2b/A/B	1a/A		1a/A	2b/A/B	1a/A	5/B
Earlier detection of patients’ problems.					1a/A				1a/A			1a/A		1a/A		1a/A	
Improves the access to services and provision of care.	1b/A		1b/A	1b/A	2b/A/B		1b/A		2b/A/B	2b/A/B	2b/A/B	2b/A/B		1b/A	2b/A/B	1b/A	5/B
Reduces waiting list.	1b/A		1b/A	1b/A			1b/A							1b/A		1b/A	
Greater satisfaction of patients’ relatives.						3b/B	3b/B										
Better team work coordination		5/B											5/B	5/B		5/B	
Improve interaction/relationship with chronic patients.		5/B											5/B	5/B		5/B	
Supports sustainability of the services.			5/B	5/B	5/B		5/B		5/B	5/B			5/B	5/B		5/B	
Effectiveness from the patients and the organisation’s perspective.																5/B	
Greater balance between needs and resources.																	5/B
Increase the capacity of problem-solving.										5/B		5/B			5/B	5/B	5/B
Care provision practice requires a complexity that justifies an advanced or specialised practice.																5/B	

Evidence Level: 1a: Systematic review (with homogeneity) or randomised control trials, 1b: Individual Randomised control trials (with narrow confidence interval), 1c: Efficiency demonstrated by clinical practice, 2b: individual cohort study (including low quality Randomised control trials; e.g., <80% follow-up), 3b: individual case-control Study, 5: expert opinion without explicit critical appraisal, or based on physiology, bench research or “first principles”; Degree of Recommendation: A: consistent level 1 studies, B: consistent level 2 or 3 studies or extrapolations from level 1 studies.
